# Colistin resistance dynamics in *Pseudomonas aeruginosa* under biofilm and planktonic growth

**DOI:** 10.1128/aac.00421-25

**Published:** 2025-09-03

**Authors:** Angie C. Alarcon Rios, Laura B. Zwep, Bastienne Vriesendorp, Catherijne A. J. Knibbe, Oana Ciofu, Apostolos Liakopoulos, Linda B. S. Aulin, Daniel E. Rozen, J. G. Coen van Hasselt

**Affiliations:** 1 Leiden Academic Centre for Drug Research, Leiden University 4496, Leiden, the Netherlands; 2 Institute of Biology, Leiden University 4496, Leiden, the Netherlands; 3 Department of Immunology and Microbiology, Costerton Biofilm Center, University of Copenhagen 4321, Copenhagen, Denmark; 4 Microbiology, Department of Biology, Utrecht University 8125, Utrecht, the Netherlands; 5 Centre for Human Drug Research 107575 , Leiden, the Netherlands; University of Fribourg, Fribourg, Switzerland

**Keywords:** *Pseudomonas aeruginosa*, evolution, colistin, biofilm, planktonic, antimicrobial resistance, population analysis profiling, phenotypic heterogeneity

## Abstract

*Pseudomonas aeruginosa* is a major pathogen in chronic biofilm-associated lung infections, particularly in patients with cystic fibrosis. Colistin is commonly used to treat these infections, although there is little understanding of how resistance evolves when cells are grown within biofilms. The current study compared the phenotypic dynamics and genetic adaptations of colistin resistance between planktonic and biofilm-grown *P. aeruginosa*. Using an *in vitro* experimental evolution approach, we passaged planktonic and biofilm cultures over 10 days under static or progressively increasing colistin concentrations. Population analysis profiling was performed daily to track resistance dynamics and heterogeneity. Whole-genome sequencing was conducted on evolved lineages. Biofilm-grown populations exhibited significantly slower resistance rates than planktonic cultures, particularly under treatments above 0.5 mg/L (1×MIC). Despite this initial delay, both biofilm- and planktonic cultures ultimately evolved similar frequencies of resistant subpopulations. Genetically, we observed shared mutations in canonical colistin resistance determinants such as *phoQ* and *qseC*. We also identified growth-mode-specific patterns: *oprH* mutations were primarily found in biofilm-evolved populations, while *nfeD* mutations were pervasive in planktonic cultures but rare in biofilms. Taken together, our results provide key insights into the role of biofilm in shaping the evolutionary trajectories of colistin resistance evolution in *P. aeruginosa*.

## INTRODUCTION

*Pseudomonas aeruginosa* is an opportunistic pathogen frequently associated with chronic respiratory infections (1, 2). Over 80% of adult cystic fibrosis (CF) patients are colonized with this pathogen (3, 4). Chronic *P. aeruginosa* infections are difficult to treat in part due to its ability to form biofilms (5, 6), which are associated with enhanced tolerance to antimicrobial agents (7). In this context, colistin is a key antibacterial agent used to treat chronic biofilm-associated *P. aeruginosa* infections (8, 9). Resistance against colistin is increasingly reported (10, 11). As such, understanding how *de novo* colistin resistance can develop during treatment of biofilm-associated infections in *P. aeruginosa* is highly relevant.

Biofilms are well known to have an increased level of resilience to antibiotics compared to planktonic bacteria. However, phenotypic and genotypic differences in the evolution of antibiotic resistance between biofilm and planktonic growth modes are less well understood. Biofilms create a structured microenvironment characterized by spatial gradients in nutrients, oxygen, and antibiotics, which enable diversification of bacterial subpopulations resulting in reduced antibiotic sensitivity. Additionally, spatial structures in biofilms may allow resistance mutations to persist that would otherwise be eliminated by strong selective sweeps in exposed planktonic populations (12, 13). Overall, the biofilm growth mode has the potential to alter antibiotic resistance dynamics and mutation fixation (14–16). While colistin resistance in planktonic populations is well characterized, it remains unclear to what extent *P. aeruginosa* growth in biofilms could lead to differences in resistance evolution from a phenotypic and genetic perspective. Addressing this gap is essential for understanding the dynamics of antimicrobial resistance during prolonged antibiotic exposures, to further guide antimicrobial treatment strategies (17, 18).

Here, we compared how static and progressively increasing colistin concentrations influence resistance evolution in biofilm and planktonic cultures of *P. aeruginosa*. We examined the rate and frequency of resistance emergence and characterized the resistance mutations of evolved strains using whole-genome sequencing. This approach allowed to assess how different drug exposure regimens impact resistance development, providing insights into biofilm-specific evolutionary responses to colistin.

## MATERIALS AND METHODS

### Experimental design

**Fig 1:**
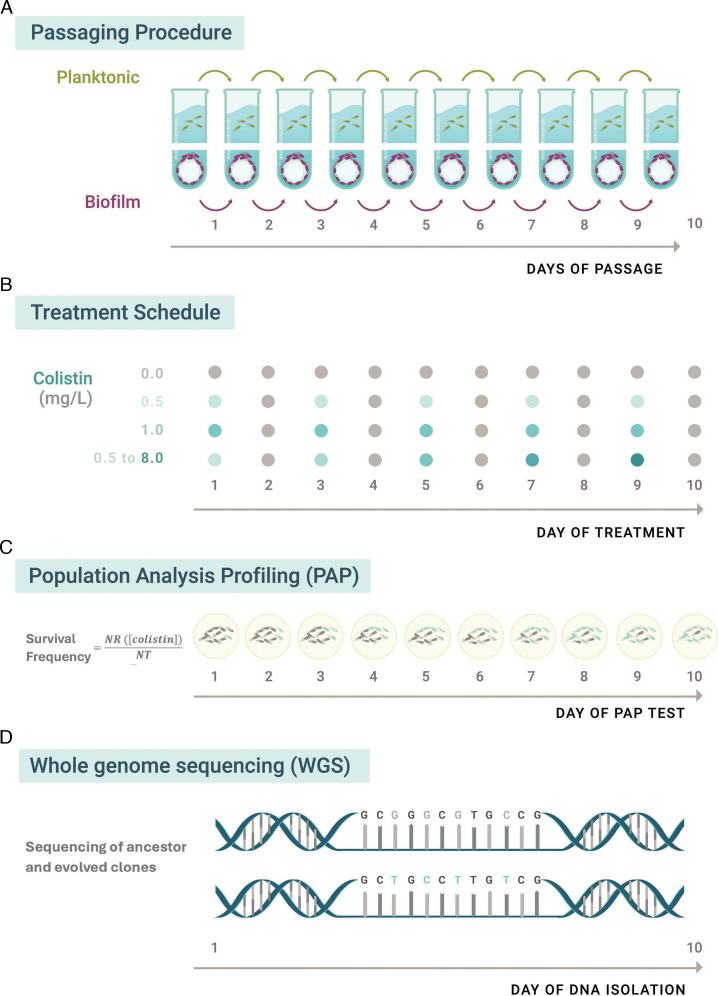
Experimental design and workflow. (**A**) Serial passaging of planktonic and biofilms evolved independently by a daily aliquot/bead passage over 10 days. (**B**) Intermittent colistin experimental treatment conditions during evolution: 0 mg/L (no treatment, gray), 0.5 mg/L, (static, light blue), 1 mg/L, (static , blue), and 0.5–8 mg/L, (increasing, dark blue) at days 1, 3, 5, 7, and 9. (**C**) Daily population analysis profiling (PAP) tests were performed to determine the survival frequency in each evolved population. (**D**) Whole-genome sequencing analysis was performed to the ancestral PAO1 strain as well as representative resistant clones from the last day of evolution.

To study colistin resistance dynamics in *P. aeruginosa* in biofilm and planktonic growth, evolution experiments were performed in synthetic CF medium (SCFM) (19) under different static and progressively increasing colistin concentrations over 10 days. Resistance was tracked using population analysis profiling (PAP) (20). Evolved populations were defined as resistant when survival frequencies exceeded 0.1%. Representative resistant clones from all evolved populations were sequenced to identify growth mode- and treatment-specific differences after experimental evolution. A schematic of the approach is shown in Fig. 1.

#### Ancestral strain and media

The *P. aeruginosa* PAO1 derivative XEN41 (21) hereafter referred to as PAO1 was used as the ancestral strain for the evolution experiment. Colistin sulfate (Cayman Chemical) was used to prepare fresh antibiotic stock solutions. All experiments were carried out using SCFM as previously described (19). Briefly, salts for M9 media buffer and individual amino acids were kept at concentrations ranging from 0.0036 to 2.1 mM and resuspended in Milli-Q water. Aspartate and diethylenetriamine pentaacetate were resuspended in 0.1 mM sodium hydroxide (NaOH) and glutamate in 0.01 mM (NaOH). The base medium was supplemented with additional trace metals including diethylenetriamine pentaacetic acid, zinc sulfate, boric acid, manganese chloride, cobalt chloride, copper sulfate, ammonium heptamolybdate, as well as BME vitamin. Phosphate-buffered saline (PBS) was used in all rinsing steps and serial dilutions.

#### Phenotypic characterization of ancestral strain

Growth dynamics and colistin minimum inhibitory concentration (MIC) for PAO1 (0.5 mg/L) were determined by a modified broth microdilution method following EUCAST guidelines (22). Cultures were incubated with shaking at 150 rpm. Optical density at 600 nm (OD_600_) measurements were taken hourly in a microplate reader (BMG Fluostar Omega) over a 24-h period in triplicate.

#### Biofilm and planktonic experimental evolution procedure

All experiments started with a 24 h liquid culture inoculated from a single colony of PAO1, prepared as biological replicates. For the passaging of both biofilm and planktonic cultures, 10^7^ cells were transferred each day (Fig. 1A). For planktonic cultures, aliquots of 10^7^ cells were determined for each transfer through linear regression analysis of the colony-forming units (CFU) in relation to OD_600_.(Fig. S1). For biofilms, 7 mm beads were used to cultivate surface-adherent biofilms for 24 h to reach a maximum carrying capacity of ~1 × 10^7^ CFU/bead (Fig. S2). At each passage, a single 7 mm colonized bead, incubated for 24 h (~10⁷ CFU/bead) was rinsed with PBS and transferred into fresh SCFM medium containing three sterile 7 mm beads. The beads follow a “bead to bead” cycle of *P. aeruginosa* dispersal, colonization, and biofilm growth at every passage, as implemented previously (14). While precise doubling times were not measured, we estimate that planktonic populations underwent ~6–8 generations per day (~60–80 total), while biofilm populations underwent ~3–5 per day (~30–50 total) due to spatial constraints and the bead-to-bead transfer method. Beads with two different colors were used to identify freshly colonized beads. During each passage, colonized beads were rinsed with PBS and passed into fresh SCFM media containing new sterile beads, following a “bead to bead” cycle of *P. aeruginosa* dispersal, colonization, and biofilm growth at every passage (14). During evolution, planktonic and biofilm cultures were incubated at 37°C in a cell culture roller drum (Fisher Scientific) at 18 rotations per minute. Daily samples of 1 × 10^7^ CFU from each culture were collected as aliquots (planktonic) or from beads (biofilm). The cells were harvested from beads by sonicating the rinsed bead (Misonix Ultrasonic Liquid Processor) for 3 min at 20°C with 1 mL of PBS. All samples were stored with 80% glycerol at −80°C for phenotypic assays and DNA isolation.

#### Colistin treatments during evolution

Three colistin concentration conditions were studied: (i) static 0.5 mg/L (1×MIC), (ii) static 1 mg/L (2× MIC), and (iii) a progressively increasing treatment, where the colistin concentration doubled every 48 h, starting at 0.5 mg/L and reaching 8 mg/L (16×MIC). Bacterial populations were exposed to colistin intermittently on days 1, 3, 5, 7, and 9, alternating every 24 h with colistin-free medium to allow for population recovery and expansion. Parallel controls without colistin were also passaged (Fig. 1B). Each treatment included three biological replicates.

#### Population analysis profiling

To capture shifts in resistance dynamics and population heterogeneity over time, we employed daily PAP, a method capable of detecting low-frequency resistant subpopulations andquantifying survival and resistance fractions across a range of colistin concentrations. PAP was performed by inoculating ~10^6^ CFU from each archived sample into 25 mL SCFM without colistin. Cultures were then incubated for 24 h at 37°C, after which dilutions were subsequently plated on Mueller Hinton (MH) agar plates containing colistin at 4, 8, 16, and 32 mg/L as well as on drug-free plates after incubation for 24 h at 37°C. CFU were quantified with an automatic colony counting system (Acolyte, Synbiosis). The upper limit of quantification was established at 10⁷ CFU/mL (100 colonies at 10⁻⁶ dilution) and the lower limit at 10 CFU/ml (10 colonies at 10⁰ dilution). The fraction of resistant cells (F_R_) was calculated using the ratio of the number of resistant (N_R_) cells surviving at different concentrations of colistin (4, 8, 16, and 32 mg/L) in PAP, over the total number of cells on the PAP plate with no colistin (N_T_) (Eq. 1):


(1) $$F_{R}\;([colistin])\;=\frac{N_{R}\;([colistin])}{N_{T}}$$

The stability of resistance in evolved mutants after 10 days of passaging was tested in all evolved populations by serially passaging 100 µL aliquots of each mutant sample for 4 days without colistin, after which the PAP assay was repeated.

#### DNA isolation and sequencing

To harvest isolates for sequencing, we used the replica plating technique to identify and distinguish coexisting mutants with different levels of resistance on the final day of the evolution experiment (23). Initially, terminal samples from all conditions were cultured in liquid media and plated on colistin-free MH plates, which were incubated for 24 h at 37°C. Using a sterile velvet, the master plates were replicated onto MH plates containing colistin at 8 and 32 mg/L, matching the concentrations used in our phenotypic PAP assays. After incubating these drug-containing plates, resistant colonies were selected via replica plating from drug-free master plates onto colistin-containing plates (8 or 32 mg/L). This approach enabled us to systematically identify and select bacterial colonies with a specific resistance profile. We randomly selected three colonies for each experimental condition. These colonies were selected, cultured, and expanded for DNA isolation. The ancestral PAO1 strain was similarly processed by culturing an aliquot of 10⁷ CFU/mL in 25 mL of antibiotic-free media for 24 h before DNA extraction (Blood and Tissue DNA Isolation Kit, Qiagen). Generation of paired-end sequence reads was performed by BaseClear B.V. (Leiden, NL) with an Illumina system. FASTQ read sequence files were generated using bcl2fastq version 2.20 (Illumina). The initial quality assessment was based on data passing the Illumina Chastity filtering. Subsequently, reads containing PhiX control signal were removed using an in-house filtering protocol established by BaseClear B.V. In addition, reads containing (partial) adapters were clipped (up to a minimum read length of 50 bp). The second quality assessment was based on the remaining reads using the FASTQC quality control tool version 0.11.8. All samples had a quality score above 30, which represents a 1 in 1,000 probability of incorrect base calling and 99.9% inferred base call accuracy.

### Data analysis

#### Rate of resistance development

To track and compare resistance rates over time, we defined a threshold to determine when *P. aeruginosa* first exhibited resistance during evolution. For each biological replicate, the time to resistance was recorded as the passage day on which this threshold was exceeded for the second timeto reduce influence of biological or measurement variation. A survival threshold of 0.1% (10⁻³) was selected to define resistance, based on empirical testing of multiple thresholds and evaluation using Kaplan-Meier and variance analyses. The threshold was empirically selected to: (i) ensure sufficient observations that exceed the threshold and (ii) to identify meaningful variability in resistance timing to allow treatment-specific comparisons. If the threshold was set too low (e.g., survival frequencies of 5 × 10⁻⁸ to 1 × 10⁻⁶), this would result in rapid detection of resistance across all populations, reducing the ability to differentiate resistance rates between experimental conditions. Meanwhile, higher thresholds (e.g., 0.01–1) led to fewer observed events but greater variability in the timing of resistance detection, making it easier to differentiate between populations and conditions and interpret colistin resistance evolution dynamics.

Statistical analyses were performed to assess differences in the time required for resistance to evolve across experimental conditions. A three-way ANOVA was conducted to determine the effects of growth mode (biofilm and planktonic), experimental colistin treatments (0 mg/L [no treatment], 0.5 mg/L [static], 1 mg/L [static], and 0.5–8 mg/L [increasing]) and PAP concentrations (4, 8, 16, and 32 mg/L) as fixed factors. Interaction terms were included to assess whether time to resistance varied with growth mode, experimental colistin treatment, and PAP. Significance was determined using an alpha threshold of 0.05. When growth conditions showed significant differences in ANOVA analysis, Tukey honest significant difference (HSD) test was performed on multiple pairwise comparisons and all interaction effects related to it. Significance was determined using an alpha threshold of 0.05.

#### Frequency of resistance

To determine and compare the final resistance frequency at day 10, a three-way ANOVA was performed using the log of the survival frequency for biofilm and planktonic (growth mode), colistin experimental treatments (0 mg/L [no treatment], 0.5 mg/L [static], 1 mg/L [static], and 0.5–8 mg/L [increasing]), and PAP colistin concentrations (4, 8, 16, and 32 mg/L) as fixed factors. Interaction terms were included to determine the effects of treatment and PAP varied across growth modes. Significance was established with an alpha threshold 0.05.

#### Genotypic analysis

*In silico* identification of genomic variants, including single-nucleotide polymorphisms (SNPs) and insertion-deletion polymorphisms, was performed using breseq v.0.38.1 with default parameters (24). As a reference genome, the short-read data from the non-evolved ancestor strain PAO1 was used to create a *de novo* assembly using SPADES v.3.15.2 (25). Gene annotation of the coding sequences was performed using Prokka v1.13.7 (26). The Illumina reads were mapped to the ancestor genome to identify mutations in the experimentally evolved strains. Analyses were carried out on the Dutch national e-infrastructure with the support of SURF Cooperative, and on the Academic Leiden Interdisciplinary Cluster Environment provided by Leiden University.

To identify SNPs in genes potentially influenced by the experimental conditions, and consequently relevant for colistin resistance development, we conducted a partial least squares discriminant analysis (PLS-DA) on the reads obtained from the mapped reads from the SNP analysis. PLS-DA is a statistical method employed to link categorical outcomes with high-dimensional input data, such as sequencing data, by effectively reducing the dimensionality to optimally distinguish between the various categories (27). The PLS-DA outcomes were growth mode (biofilm or planktonic), colistin treatment concentrations during evolution (0, 0.5, 1, and 8 mg/L) and the CFU/mL from the PAP plates (8 and 32 mg/L), where isolates were harvested for sequencing analysis. PLS-DA was applied to each of these outcomes identifying mutations not present in the ancestor PAO1. This analysis yielded a variable importance in projection (VIP) score for each gene. This score indicated how important a gene was among all populations in the different evolutionary conditions. The genes with the highest VIP score (>1.5) were of interest for their respective outcome and were further explored in the literature (Table S4).

#### Software

Data visualization, statistical analyses, and model evaluation were carried out in R 4.2.2. R Studio and packages tidyverse, survival, survminer, and ggplot2 were used for all the data analysis. Experimental data and code are available on GitHub under Data R Files S1 and S2.

## RESULTS

### Colistin resistance evolution

To investigate colistin resistance dynamics in *P. aeruginosa*, we tracked shifts in survival and resistance frequencies in biofilm and planktonic-grown cells over 10 days of evolution. Populations were exposed to static colistin concentrations (0.5 and 1 mg/L) or to progressively increasing concentrations (0.5–8 mg/L). Resistance phenotypes were assessed daily through PAP profiling.

**Fig 2:**
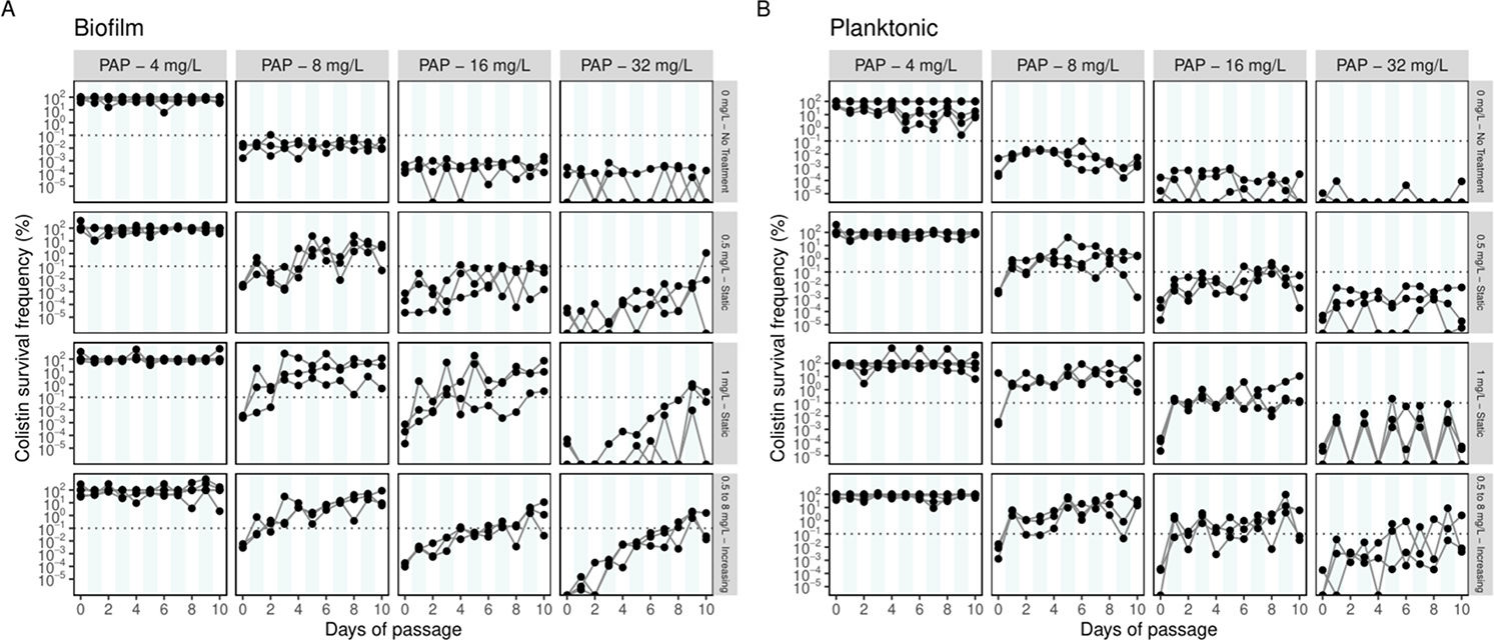
Colistin survival frequency of *P. aeruginosa* in biofilm (**A**) and planktonic (**B**) growth modes over days of passage under four experimental treatment conditions (panel rows) during evolution and different PAP colistin concentrations for population analysis profiling (PAP) assays used to assess resistance (panel columns). Dashed lines highlight the resistance frequency threshold (0.1%) for statistical comparisons of resistance rate dynamics across treatments and growth modes. Each point represents a biological replicate.

At the onset of the evolution experiments, phenotypic heteroresistance was observed in both biofilm and planktonic cultures with initial survival frequencies below 0.1% (Fig. 2). This phenotypic heteroresistance, characterized by subpopulations able to survive high concentrations of colistin despite the majority being susceptible (28), was detected across most colistin concentrations tested (8–32 mg/L). Notably, biofilm cultures exhibited larger heteroresistant subpopulations than their planktonic counterparts, indicating a potential growth-mode influence on the initial prevalence of phenotypic heterogeneity (Fig. 2A, top row).

In the absence of colistin, the survival of heteroresistant subpopulations remained stable over time for both biofilm and planktonic-grown cells (Fig. 2). By contrast, over the course of 10 days of evolution, both biofilm and planktonic subpopulations able to survive colistin were positively selected. At the terminal time point (day 10), populations exposed to static (1 mg/L) and progressively increasing (0.5–8 mg/L) colistin treatments displayed resistance frequencies exceeding 10% in PAP at 16 mg/L (Fig. 2). Resistance stability was confirmed over four additional days of passage without colistin selection.

To assess population-level changes in susceptibility, MICs were determined at the terminal time point (day 10) for all evolved populations using broth microdilution. MIC values ranged from 0.5 to 1 mg/L, representing at most a twofold increase from the ancestral MIC (0.5 mg/L). Notably, these modest MIC shifts contrast with the high-level resistance detected in PAP, where subpopulations survived up to 16–32 mg/L.

Kaplan-Meier analysis across treatments and growth modes showed that biofilms exhibited a marginally higher frequency of resistance compared to planktonic regardless of the survival threshold (Fig. S5). For the subsequent analyzes on resistance dynamics, populations with survival frequencies above a 0.1% threshold were classified as resistant.

### Rate of resistance development

**Fig 3:**
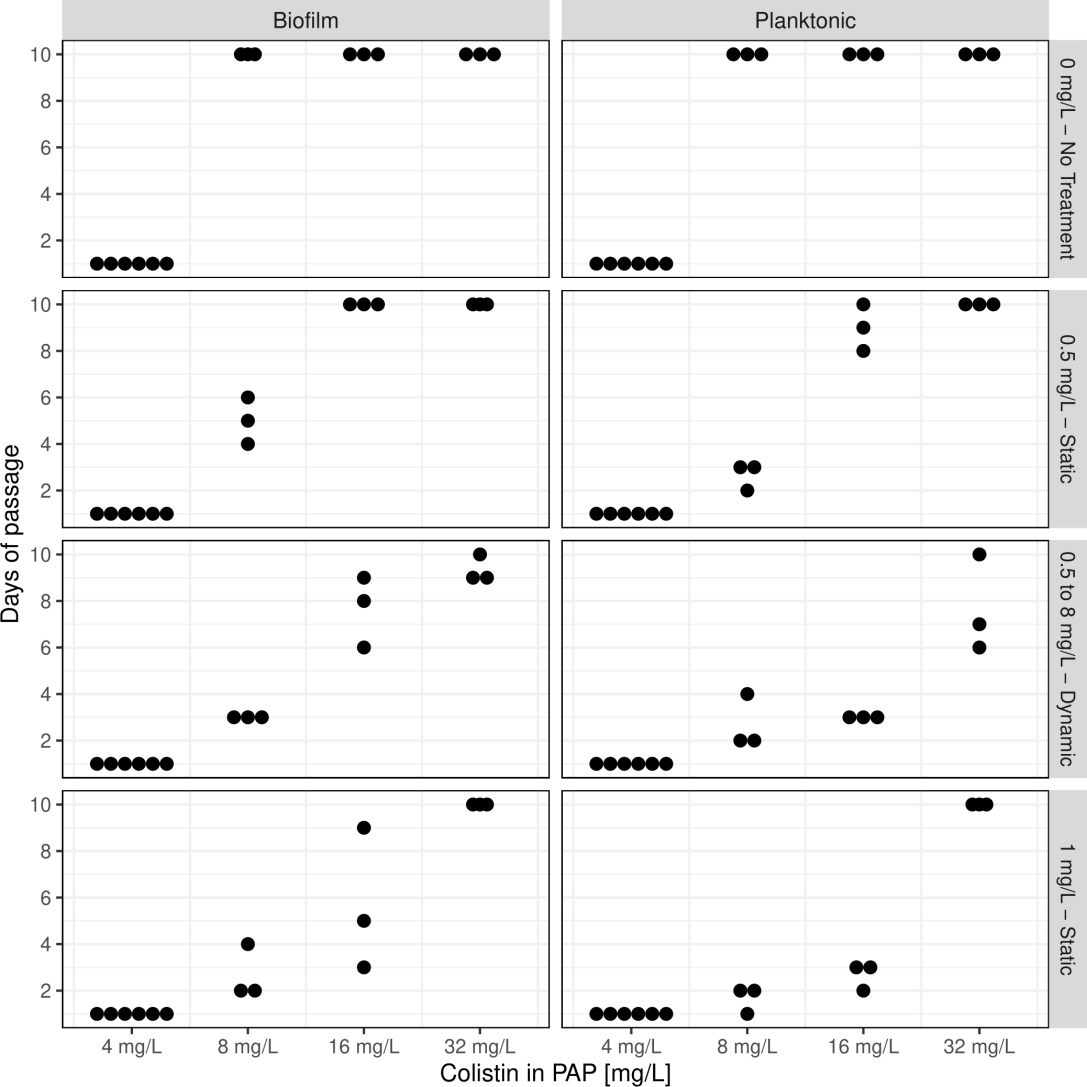
Time to reach resistance threshold (0.1% survival) for different colistin treatment conditions (panel rows) and growth modes (panel columns).

To evaluate the impact of colistin treatments on the rate of resistance development in both biofilm and planktonic growth modes, we analyzed the time to resistance emergence over 10 days of passage. Generally, planktonic-grown cells evolved resistance more rapidly than biofilm-grown cells (Fig. 3). ANOVA confirmed significant effects of growth mode, experimental colistin treatment, and PAP on time to resistance (*P* < 0.05). Significant interactions were also observed between growth modes and PAP, growth modes and colistin treatment, and PAP and colistin treatment (Table S1).

When considering the specific effects of colistin treatment at concentrations ≥1 mg/L and across PAP tests, planktonic grown cells exhibited significantly faster resistance rates compared to biofilm cultures (Table S2). This effect was absent in the no-treatment control, where both growth modes showed similar survival frequencies.

### Resistance frequency after experimental evolution

**Fig 4:**
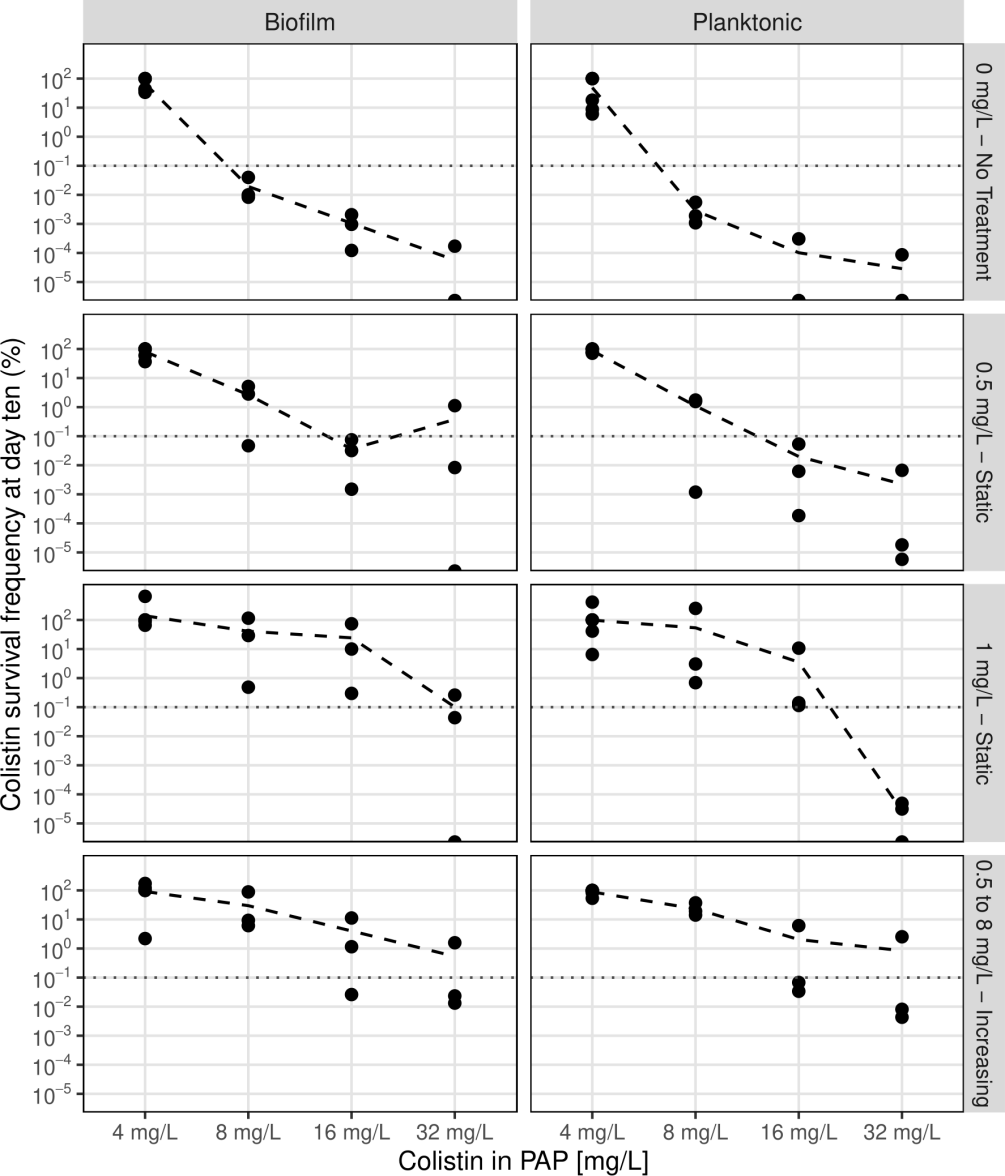
Colistin survival frequencies (%) for different resistance levels surviving assessed through population analysis (PAP) profiling, for different colistin treatment conditions (panel rows) and growth modes (panel columns). Dotted lines highlight the resistance frequency threshold (0.1%). Each point represents a biological replicate, and lines represent mean values.

Colistin resistance dynamics varied by treatment and growth mode. Figure 4 illustrates the colistin survival and resistance frequencies of biofilm and planktonic-grown populations across different treatment conditions and PAP concentrations. In the absence of colistin, both biofilm and planktonic populations exhibited survival frequencies below the resistance threshold (0.1%), despite initial differences in survival frequencies and resistance emergence rates.

Resistance frequencies significantly increased (*P* < 0.001) in all colistin-treated populations over the course of experimental evolution (Table S3). Biofilm and planktonicpopulations ultimately evolved comparable frequencies of resistant subpopulations at the terminal time point (day 10), with no statistically significant difference in resistance (survival frequency>0.1%) was identified (Table S3). In populations exposed to static colistin treatments below 1 mg/L, resistance remained higher in biofilms at 16 and 32 mg/L, while planktonic populations fell below this threshold. Under progressively increasing colistin concentrations (0.5–8 mg/L), resistance frequencies converged across growth modes at PAP 32 mg/L.

### Genotypic analysis of biofilm and planktonic-evolved cells

To investigate the genetic basis of colistin resistance, we obtained whole genome sequences of *P. aeruginosa* isolates from biofilm and planktonic cultures after experimental evolution. We selected representative isolates able to survive on colistin-supplemented PAP plates at concentrations of 8 or 32 mg/L. In total, we identified 45 genes with SNPs. For the biofilm cultures, 1326 SNPs were identified, while for planktonic-derived isolates 1,423 SNPs were found.

To classify genes potentially influenced by the evolutionary conditions and relevant to colistin resistance development, we conducted a PLS-DA directly on sequencing data. PLS-DA linked our categorical outcomes—colistin treatment, PAP concentrations, and growth mode—with the high-dimensional input data from the genomic sequencing. From this analysis we derived four genes: *oprH*, *nfeD*, *phoQ*, and *qseC* with high VIP scores exceeding 1.5, suggesting their contribution to different colistin treatments and growth modes during the evolution experiment (Fig. S4).

**Fig 5:**
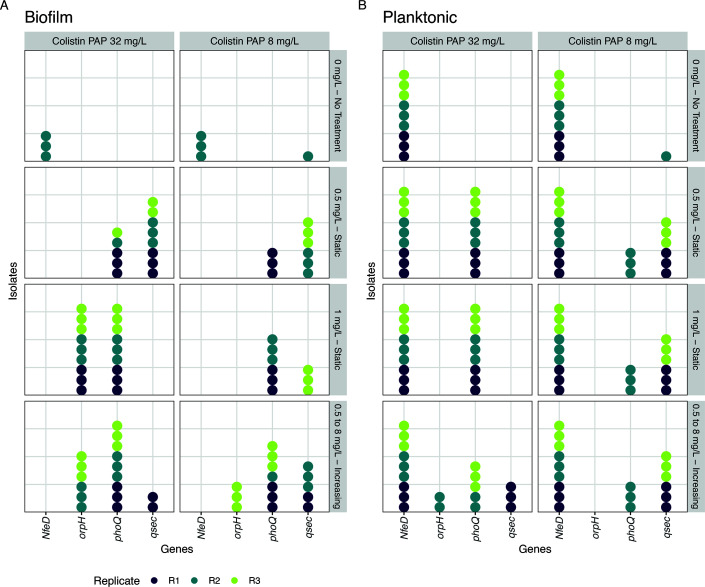
Distribution of single-nucleotide polymorphisms (SNPs) in selected key resistance-associated genes in representative isolates from all evolved populations at day 10. Each panel compares (**A**) biofilm and (**B**) planktonic populations across different colistin treatments (panel rows) during evolution surviving at colistin containing plates of 8 and 32 mg/L (panel columns). Points and colors represent the different biological replicates. Nine colonies per condition were selected from colistin-containing PAP plates at 8 and 32 mg/L.

We observed a predominance of non-synonymous mutations across multiple genes associated with colistin resistance, exhibiting varying patterns between biofilm and planktonic growth modes (Table S3). Nonsense mutations and deletions in the coding region of *oprH* were consistently detected in biofilm populations exposed to static (1 mg/L) and increasing colistin concentrations, specifically in isolates harvested from colistin PAP plates at 32 mg/L. Conversely, a hotspot of mutations in the nodulation formation efficiency protein NfeD was pervasive in planktonic populations, regardless of colistin exposure or harvest conditions. These mutations were only observed in a small subset of biofilm isolates not exposed to colistin (Fig. 5A).

We identified a parallel hotspot of mutations at the gene level in the virulence sensor histidine kinase *phoQ* gene across all colistin-treated biofilm and planktonic populations. However, the specific SNPs were not identical between replicates or treatments (Fig. S3). Similarly, point mutations and deletions in the *qseC* gene were detected across all evolutionary conditions but followed distinct patterns between growth modes. Qsec mutations were predominantly displayed by isolates from treated populations harvested from colistin PAP plates at 8 mg/L and populations exposed to increasing colistin treatment that were harvested from colistin PAP plates at 32 mg/L (Fig. 5).

## DISCUSSION

This study provides insights into colistin resistance evolution in *P. aeruginosa* during biofilm and planktonic growth. Colistin resistance was rapidly selected in both environments under varying treatment regimens. Although resistance emerged more slowly in biofilms, both lifestyles ultimately converged to similar resistance levels and frequencies under prolonged antibiotic exposure. Despite these differences, both lifestyles ultimately converged to similar resistance levels under sustained selective pressure. Whole-genome sequencing revealed parallel mutations in established colistin-resistance genes as well as distinct mutations shaped by growth mode and treatment conditions.

Biofilm-grown cells initially exhibited higher survival frequencies than planktonic populations. However, under colistin treatment, biofilm populations continuously displayed significantly slower rates of resistance (Table S1), likelydue to its structured microenvironment where mutational events, diverse metabolic and growth phases mitigate antibiotic selection enabling surviving subpopulations to coexist (7, 14, 29). In contrast, planktonic growing cells face more uniform selection pressures in their immediate environment, accelerating resistance development (14, 15) through beneficial mutations or phenotypic adaptations (30).The observed differences in resistance rateand mutational trajectories between planktonic and biofilm populations may, in part, be influenced by differences in the number of elapsed generations. Biofilm populations likely underwent fewer total generations due to growth constraints and structured dispersal. This reduced generational turnover, combined with the spatial structure inherent to biofilms, likely delayed resistance emergence and shaped distinct evolutionary trajectories from those of planktonic. Notably, these features mirror the ecological constraints faced by biofilm populations *in vivo*, particularly in chronic infections, where limited nutrient diffusion, spatial segregation, and localized stress exposure can restrict evolutionary dynamics. As such, the delayed resistance and divergent mutational patterns observed in biofilms may more closely reflect the selective environment encountered during real-world infections. These evolutionary differences could help explain the delayed resistance emergence and distinct genetic patterns observed in biofilms compared to planktonic populations.

At the onset of the evolution experiment, biofilm-grown cells exhibited higher survival frequencies than planktonic populations (Fig. 2). At day 10 resistance frequencies converged across growth modes, under colistin concentrations above 0.5 mg/L (Ancestor MIC) reaching ~8% at PAP 32 mg/L. This convergence highlights colistin’s ability to overcome the protective effects of biofilms, probably due to its mode of action. Colistin disrupts bacterial membranes by binding to lipopolysaccharides (LPS) and displacing stabilizing ions, permeabilizing both outer and inner membranes (31). Its hydrophilic and hydrophobic structure enables penetration into biofilm matrices, targeting even metabolically inactive cells (32). Our findings suggest that colistin is bactericidal for biofilm-grown cells when concentrations are sufficient to bypass the protection offered by the biofilm and adaptive resistance, resulting in similar resistance for both planktonic and biofilm growth modes. Of note, in addition to PAP assays, we also measured MICs for all terminal mutants, which ranged from 0.5 to 1 mg/L. In contrast, PAP detected subpopulations surviving 16–32 mg/L. This highlights MIC’s limitation in capturing minority variants and supports our use of PAP to resolve resistance dynamics.

Whole-genome sequencing revealed growth mode- and treatment-specific genetic adaptations associated with colistin resistance (Table S3). In biofilm populations, mutations in *oprH*, which encodes the inducible outer membrane protein H1 (33), were common but rarely observed in planktonic populations. Nonsense mutations and deletions dominated, suggesting adaptation through loss of function of the H1 protein, providing a selective advantage toward resistance in *P. aeruginosa* (34) by interfering with colistin uptake (35). Notably, co-occurrence of *phoQ* and *oprH* mutations was observed almost exclusively in biofilm isolates, suggesting a potential functional relationship. These could be attributed to their transcriptional linkage, or the proximity of their coding regions within a common operon, where mutations in *phoQ* might influence the regulation or expression of *oprH* (36). Given their shared roles in outer membrane adaptation under cationic stress, this co-selection may represent a biofilm-specific adaptive mechanism of colistin resistance evolution. Similar genetic adaptations have been identified in *P. aeruginosa* isolates from the CF lung, where chronic infection fosters both biofilm formation and the co-expression of *phoQ* and *oprH* as part of an adaptive survival strategy (37).

Mutations in *nfeD* were pervasive in planktonic populations across all conditions, including the no-colistin control, but absent in colistin-treated biofilms. *nfeD* is linked to protease activation and membrane integrity under cationic stress (38), crucial processes for planktonic growth and survival. n*feD* interactions with flotilins have been linked to morphological defects, motility loss, and impaired biofilm formation (39), which may explain their absence, as these mutations cause detrimental effects on biofilm growth. The consistent presence of n*feD* mutations in planktonic populations suggests it plays an active role in growth-specific adaptation that supports membrane remodeling and increases stress tolerance (38) to perpetuate planktonic growth independently of antibiotic selection.

Mutations in *phoQ*, a well-established gene associated with colistin resistance, were identified across both biofilm and planktonic populations. The PhoP/PhoQ two-component system, typically activated under acidic conditions, modifies outer membrane LPS (40, 41), reducing affinity for positively charged colistin molecules and hindering uptake (42). The mutations observed in *phoQ* varied across conditions and biological replicates but occurred in the same genomic region, suggesting independent mutational events converging on a common adaptive target. These findings are consistent with previous studies, reinforcing *phoQ*’s conserved role in colistin resistance regardless of growth mode (40, 41).

Mutations in *qseC* were nearly ubiquitous in isolates harvested from colistin PAP plates at 8 and 32 mg/L in both biofilm and planktonic growth modes. This two-component system (43) responds to cationic stress, triggering regulatory cascades (44) involving PmrA and QseB, which modify LPS and enhance resistance in *P. aeruginosa* (45), *Escherichia coli* (46), and *Salmonella enterica* (47).

Interestingly, our sequencing did not detect mutations in *pmrB*, a two-component system sensor kinase frequently associated with colistin resistance via LPS modification (48). This absence may reflect condition-specific selection pressures or redundancy in resistance mechanisms, where mutations in *phoQ*, *qseC*, or *oprH* may have provided sufficient adaptive benefit under our experimental conditions.

It is important to note that the PLS-DA VIP derived scores indicate association strength, not causality. While high-scoring genes like *phoQ* support model validity, others such as *nfeD* may reflect growth-mode-specific adaptations rather than direct resistance roles. These associations guide interpretation but require functional follow-up.

Our findings highlight the dual nature of resistance evolution in *P. aeruginosa*, with conserved pathways like *phoQ* and *qseC* driving adaptation across environments, while growth-mode-specific mutations such as those in *oprH* and *nfeD* highlight the influence of biofilm structure and planktonic dynamics. This study demonstrates the importance of understanding both universal and niche-specific mechanisms of resistance.

Daily PAP assays revealed heterogeneity in the adaptive pathways in different experimental populations. While this may be influenced by clonal interference—where multiple resistant subpopulations with competing beneficial mutations emerge simultaneously—the evidence remains inconclusive. These competing dynamics could have slowed down the fixation of fully resistant phenotypes, delaying the establishment of widespread resistance within the population. These findings highlight the complexity of resistance evolution, particularly in structured environments like biofilms, where spatial heterogeneity and a sustained diversity of adaptations might influence resistance trajectories (49).

Future studies using diverse sets of clinical strains and more complex models, such as dynamic flow-cell biofilm reactors or animal models of chronic infections, are necessary to refine our understanding of biofilm-associated resistance in *P. aeruginosa*. Despite these limitations, this study addresses critical gaps in understanding growth-mode-specific resistance dynamics. Colistin accelerated resistance development across both planktonic and biofilm growth modes, with resistance rates significantly slower in biofilms but ultimately converging under high concentrations. This convergence suggests that colistin’s ability to penetrate biofilms and target metabolically inactive cells is sufficient to overcome the protective effects of the biofilm structure.

### Conclusion

This study reveals differences and similarities in colistin resistance between *P. aeruginosa* biofilm and planktonic populations. Biofilms exhibited a delayed onset of resistance emergence as compared to planktonic populations, likely due to their protective structure. However, under high colistin concentrations, both growth modes converged to similar resistance frequencies. Genetic analysis revealed growth-mode specific mutational patterns, despite modifications in common colistin resistance-associated targets. Mutations in *nfeD* were predominantly found in planktonic populations, suggesting growth-mode-specific membrane adaptations. In biofilms, co-occurring mutations in *phoQ* and *oprH* suggested synergistic outer membrane adaptations, likely enhancing colistin tolerance. Conversely, mutations in *phoQ* and *qseC* were shared across treatments, reinforcing their role as conserved colistin resistance targets, yet with distinct mutational pathways across biofilm and planktonic growth modes. Taken together, these findings emphasize that while biofilms influence the trajectory of resistance evolution and promote colistin tolerance, the genetic pathways leading to final resistance frequencies are parallel to those observed in planktonic populations. Understanding resistance evolution dynamics across different growth modes is essential for the development of comprehensive approaches that consider the complexity of resistance mechanisms to effectively manage and mitigate colistin resistance evolution.

## SUPPLEMENTAL MATERIAL

Supplemental material aac.00421-25-s0001.pdf Tables S1 to S4; Fig. S1 to S6.

ASM does not own the copyrights to Supplemental Material that may be linked to, or accessed through, an article. The authors have granted ASM a non-exclusive, world-wide license to publish the Supplemental Material files. Please contact the corresponding author directly for reuse.

## References

[1] CDC. 2019. Antibiotic resistance threats in the United States. Available from: https://www.cdc.gov/drugresistance/pdf/threats-report/2019-ar-threats-report-508.pdf

[2] Tornimbene B, Eremin S, Escher M, Griskeviciene J, Manglani S, Pessoa-Silva CL. 2018. WHO global antimicrobial resistance surveillance system early implementation 2016-17. Lancet Infect Dis 18:241–242. doi:10.1016/S1473-3099(18)30060-4 29396007

[3] Pressler T, Bohmova C, Conway S, Dumcius S, Hjelte L, Høiby N, Kollberg H, Tümmler B, Vavrova V. 2011. Chronic Pseudomonas aeruginosa infection definition: EuroCareCF working group report. J Cyst Fibros 10:S75–S78. doi:10.1016/S1569-1993(11)60011-8 21658646

[4] Govan JR, Deretic V. 1996. Microbial pathogenesis in cystic fibrosis: mucoid Pseudomonas aeruginosa and Burkholderia cepacia. Microbiol Rev 60:539–574. doi:10.1128/mr.60.3.539-574.1996 8840786 PMC239456

[5] Ciofu O, Moser C, Jensen PØ, Høiby N. 2022. Tolerance and resistance of microbial biofilms. Nat Rev Microbiol 20:621–635. doi:10.1038/s41579-022-00682-4 35115704

[6] Bjarnsholt T. 2013. The role of bacterial biofilms in chronic infections. APMIS 121:1–58. doi:10.1111/apm.12099 23635385

[7] Thöming JG, Häussler S. 2022. Pseudomonas aeruginosa is more tolerant under biofilm than under planktonic growth conditions: a multi-isolate survey. Front Cell Infect Microbiol 12:851784. doi:10.3389/fcimb.2022.851784 35295755 PMC8920030

[8] Falagas ME, Kasiakou SK, Saravolatz LD. 2005. Colistin: the revival of polymyxins for the management of multidrug-resistant gram-negative bacterial infections. Clin Infect Dis 40:1333–1341. doi:10.1086/429323 15825037

[9] WHO. 2019. Critically important antimicrobials for human medicine 6th revision 2018 ranking of medically important antimicrobials for risk management of antimicrobial resistance due to non-human use. In WHO Advisory Group on Integrated Surveillance of Antimicrobial Resistance (AGISAR). Vol. 6.

[10] Andrade FF, Silva D, Rodrigues A, Pina-Vaz C. 2020. Colistin update on its mechanism of action and resistance, present and future challenges. Microorganisms 8:1716. doi:10.3390/microorganisms8111716 33147701 PMC7692639

[11] Wang R, van Dorp L, Shaw LP, Bradley P, Wang Q, Wang X, Jin L, Zhang Q, Liu Y, Rieux A, Dorai-Schneiders T, Weinert LA, Iqbal Z, Didelot X, Wang H, Balloux F. 2018. The global distribution and spread of the mobilized colistin resistance gene mcr-1. Nat Commun 9:1179. doi:10.1038/s41467-018-03205-z 29563494 PMC5862964

[12] Poltak SR, Cooper VS. 2011. Ecological succession in long-term experimentally evolved biofilms produces synergistic communities. ISME J 5:369–378. doi:10.1038/ismej.2010.136 20811470 PMC3105725

[13] Ahmed MN, Abdelsamad A, Wassermann T, Porse A, Becker J, Sommer MOA, Høiby N, Ciofu O. 2020. The evolutionary trajectories of P. aeruginosa in biofilm and planktonic growth modes exposed to ciprofloxacin: beyond selection of antibiotic resistance. NPJ Biofilms Microbiomes 6:28. doi:10.1038/s41522-020-00138-8 32709907 PMC7381665

[14] Scribner MR, Santos-Lopez A, Marshall CW, Deitrick C, Cooper VS. 2020. Parallel evolution of tobramycin resistance across species and environments. mBio 11:e00932-20. doi:10.1128/mBio.00932-20 32457248 PMC7251211

[15] Santos-Lopez A, Marshall CW, Scribner MR, Snyder DJ, Cooper VS. 2019. Evolutionary pathways to antibiotic resistance are dependent upon environmental structure and bacterial lifestyle. Elife 8:e47612. doi:10.7554/eLife.47612 31516122 PMC6814407

[16] Coenye T, Bové M, Bjarnsholt T. 2022. Biofilm antimicrobial susceptibility through an experimental evolutionary lens. NPJ Biofilms Microbiomes 8:82. doi:10.1038/s41522-022-00346-4 36257971 PMC9579162

[17] Döring G, Flume P, Heijerman H, Elborn JS, Consensus Study Group. 2012. Treatment of lung infection in patients with cystic fibrosis: current and future strategies. J Cyst Fibros 11:461–479. doi:10.1016/j.jcf.2012.10.004 23137712

[18] Spellberg B, Bartlett JG, Gilbert DN. 2013. The future of antibiotics and resistance. N Engl J Med 368:299–302. doi:10.1056/NEJMp1215093 23343059 PMC3617123

[19] Palmer KL, Aye LM, Whiteley M. 2007. Nutritional cues control Pseudomonas aeruginosa multicellular behavior in cystic fibrosis sputum. J Bacteriol 189:8079–8087. doi:10.1128/JB.01138-07 17873029 PMC2168676

[20] Andersson DI, Nicoloff H, Hjort K. 2019. Mechanisms and clinical relevance of bacterial heteroresistance. Nat Rev Microbiol 17:479–496. doi:10.1038/s41579-019-0218-1 31235888

[21] Fink D, Romanowski K, Valuckaite V, Babrowski T, Kim M, Matthews JB, Liu D, Zaborina O, Alverdy JC. 2011. Pseudomonas aeruginosa potentiates the lethal effect of intestinal ischemia-reperfusion injury: the role of in vivo virulence activation. J Trauma 71:1575–1582. doi:10.1097/TA.0b013e31821cb7e5 22002612 PMC3245766

[22] European Committee for Antimicrobial Susceptibility Testing (EUCAST) of the European Society of Clinical Microbiology and Infectious Diseases (ESCMID). 2003. Determination of minimum inhibitory concentrations (MICs) of antibacterial agents by broth dilution. Clin Microbiol Infect 9:ix–xv. doi:10.1046/j.1469-0691.2003.00790.x

[23] Lederberg J, Lederberg EM. 1952. Replica plating and indirect selection of bacterial mutants. J Bacteriol 63:399–406. doi:10.1128/jb.63.3.399-406.1952 14927572 PMC169282

[24] Deatherage DE, Barrick JE. 2014. Identification of mutations in laboratory-evolved microbes from next-generation sequencing data using breseq. Methods Mol Biol 1151:165–188. doi:10.1007/978-1-4939-0554-6_12 24838886 PMC4239701

[25] Prjibelski A, Antipov D, Meleshko D, Lapidus A, Korobeynikov A. 2020. Using SPAdes de novo assembler. Curr Protoc Bioinformatics 70:e102. doi:10.1002/cpbi.102 32559359

[26] Seemann T. 2014. Prokka: rapid prokaryotic genome annotation. Bioinformatics 30:2068–2069. doi:10.1093/bioinformatics/btu153 24642063

[27] Barker M, Rayens W. 2003. Partial least squares for discrimination. J Chemom 17:166–173. doi:10.1002/cem.785

[28] El-Halfawy OM, Valvano MA. 2015. Antimicrobial heteroresistance: an emerging field in need of clarity. Clin Microbiol Rev 28:191–207. doi:10.1128/CMR.00058-14 25567227 PMC4284305

[29] Wong A, Rodrigue N, Kassen R. 2012. Genomics of adaptation during experimental evolution of the opportunistic pathogen Pseudomonas aeruginosa. PLoS Genet 8:e1002928. doi:10.1371/journal.pgen.1002928 23028345 PMC3441735

[30] Trubenová B, Roizman D, Rolff J, Regoes RR. 2022. Modeling polygenic antibiotic resistance evolution in biofilms. Front Microbiol 13:916035. doi:10.3389/fmicb.2022.916035 35875522 PMC9301000

[31] Li Z, Velkov T. 2019. Polymyxins: mode of action, p 37–54. In Li J, Nation RL, Kaye KS (ed), Polymyxin antibiotics: from laboratory bench to bedside. Springer International Publishing, Cham.

[32] Lora-Tamayo J, Murillo O, Ariza J. 2019. Clinical use of colistin in biofilm-associated infections, p 181–195. In Li J, Nation RL, Kaye KS (ed), Polymyxin antibiotics: from laboratory bench to bedside. Springer International Publishing, Cham. 10.1007/978-3-030-16373-0_13 31364079

[33] Bell A, Hancock RE. 1989. Outer membrane protein H1 of Pseudomonas aeruginosa: purification of the protein and cloning and nucleotide sequence of the gene. J Bacteriol 171:3211–3217. doi:10.1128/jb.171.6.3211-3217.1989 2498288 PMC210039

[34] Hanna SL, Sherman NE, Kinter MT, Goldberg JB. 2000. Comparison of proteins expressed by Pseudomonas aeruginosa strains representing initial and chronic isolates from a cystic fibrosis patient: an analysis by 2-D gel electrophoresis and capillary column liquid chromatography-tandem mass spectrometry. Microbiology (Reading) 146:2495–2508. doi:10.1099/00221287-146-10-2495 11021925

[35] Bell A, Bains M, Hancock REW. 1991. Pseudomonas aeruginosa outer membrane protein OprH: expression from the cloned gene and function in EDTA and gentamicin resistance. J Bacteriol 173:6657–6664. doi:10.1128/jb.173.21.6657-6664.1991 1938872 PMC209012

[36] Macfarlane ELA, Kwasnicka A, Ochs MM, Hancock REW. 1999. PhoP–PhoQ homologues in Pseudomonas aeruginosa regulate expression of the outer‐membrane protein OprH and polymyxin B resistance. Mol Microbiol 34:305–316. doi:10.1046/j.1365-2958.1999.01600.x 10564474

[37] Bielecki P, Komor U, Bielecka A, Müsken M, Puchałka J, Pletz MW, Ballmann M, Martins dos Santos VAP, Weiss S, Häussler S. 2013. Ex vivo transcriptional profiling reveals a common set of genes important for the adaptation of Pseudomonas aeruginosa to chronically infected host sites. Environ Microbiol 15:570–587. doi:10.1111/1462-2920.12024 23145907

[38] Butcher BG, Helmann JD. 2006. Identification of Bacillus subtilis σ^W^-dependent genes that provide intrinsic resistance to antimicrobial compounds produced by Bacilli. Mol Microbiol 60:765–782. doi:10.1111/j.1365-2958.2006.05131.x 16629676

[39] Dempwolff F, Moller HM, Graumann PL. 2012. Synthetic motility and cell shape defects associated with deletions of flotillin/reggie paralogs in Bacillus subtilis and Interplay of these proteins with NfeD proteins. J Bacteriol 194:4652–4661. doi:10.1128/JB.00910-12 22753055 PMC3415494

[40] Macfarlane ELA, Kwasnicka A, Hancock REW. 2000. Role of Pseudomonas aeruginosa PhoP-phoQ in resistance to antimicrobial cationic peptides and aminoglycosides. Microbiology (Reading) 146:2543–2554. doi:10.1099/00221287-146-10-2543 11021929

[41] Doijad SP, Gisch N, Frantz R, Kumbhar BV, Falgenhauer J, Imirzalioglu C, Falgenhauer L, Mischnik A, Rupp J, Behnke M, et al.. 2023. Resolving colistin resistance and heteroresistance in Enterobacter species. Nat Commun 14:140. doi:10.1038/s41467-022-35717-0 36627272 PMC9832134

[42] Jangir PK, Ogunlana L, Szili P, Czikkely M, Shaw LP, Stevens EJ, Yu Y, Yang Q, Wang Y, Pál C, Walsh TR, MacLean CR. 2023. The evolution of colistin resistance increases bacterial resistance to host antimicrobial peptides and virulence. Elife 12:e84395. doi:10.7554/eLife.84395 37094804 PMC10129329

[43] Breland EJ, Zhang EW, Bermudez T, Martinez CR 3rd, Hadjifrangiskou M. 2017. The histidine residue of QseC is required for canonical signaling between QseB and PmrB in uropathogenic Escherichia coli. J Bacteriol 199:e00060-17. doi:10.1128/JB.00060-17 28396353 PMC5573081

[44] Fernandez-Ciruelos B, Potmis T, Solomin V, Wells JM. 2023. Cross-talk between QseBC and PmrAB two-component systems is crucial for regulation of motility and colistin resistance in Enteropathogenic Escherichia coli. PLoS Pathog 19:e1011345. doi:10.1371/journal.ppat.1011345 38060591 PMC10729948

[45] Olaitan AO, Morand S, Rolain J-M. 2014. Mechanisms of polymyxin resistance: acquired and intrinsic resistance in bacteria. Front Microbiol 5:643. doi:10.3389/fmicb.2014.00643 25505462 PMC4244539

[46] Guckes KR, Breland EJ, Zhang EW, Hanks SC, Gill NK, Algood HMS, Schmitz JE, Stratton CW, Hadjifrangiskou M. 2017. Signaling by two-component system noncognate partners promotes intrinsic tolerance to polymyxin B in uropathogenic Escherichia coli. Sci Signal 10:eaag1775. doi:10.1126/scisignal.aag1775 28074004 PMC5677524

[47] Merighi M, Septer AN, Carroll-Portillo A, Bhatiya A, Porwollik S, McClelland M, Gunn JS. 2009. Genome-wide analysis of the PreA/PreB (QseB/QseC) regulon of Salmonella enterica serovar Typhimurium. BMC Microbiol 9:42. doi:10.1186/1471-2180-9-42 19236707 PMC2653508

[48] Michaelis C, Grohmann E. 2023. Horizontal gene transfer of antibiotic resistance genes in biofilms. Antibiotics (Basel) 12:328. doi:10.3390/antibiotics12020328 36830238 PMC9952180

[49] Harris KB, Flynn KM, Cooper VS. 2021. Polygenic adaptation and clonal interference enable sustained diversity in experimental Pseudomonas aeruginosa populations. Mol Biol Evol 38:5359–5375. doi:10.1093/molbev/msab248 34410431 PMC8662654

