# Supplementary material for: Colistin resistance dynamics in Pseudomonas aeruginosa under biofilm and planktonic growth
Source: Antimicrob Agents Chemother. 2025 Sep 3;69(10):e00421-25. doi: 10.1128/aac.00421-25 (PMC12486798; doi:10.1128/aac.00421-25)
Supplement: Supplemental material — Tables S1 to S4; Fig. S1 to S6. [file aac.00421-25-s0001.pdf]

Supplemental tables and figures

Table S1. Time to resistance ANOVA during experimental evolution between growth modes (biofilm – planktonic), colistin experimental treatments (0 mg/L – No Treatment, 0.5 mg/L- Static, 1 mg/L- Static, and 0.5 to 8 mg/L- Increasing), and PAP colistin concentrations (4 mg/L, 8 mg/L, 16 mg/L, and 32 mg/L).

	Df	Sum Sq	Mean Sq	F value	Pr(>F)	significant
**Growth Mode**	**1**	**14,7**	**14,7**	**29,4**	**5,10E-07**	*****
PAP	3	1395,3	465,1	930,189	<2E-16	*
Treatment	3	150,8	50,3	100,511	<2E-16	*
Growth Mode:PAP	3	19,5	6,5	13,033	4,05E-07	*
**Growth Mode:Treatment**	**3**	**6,8**	**2,3**	**4,511**	**0,00544**	*****
PAP:Treatment	9	246,7	27,4	54,811	<2E-16	*
**Growth Mode:PAP:Treatment**	**9**	**20,7**	**2,3**	**4,589**	**5,60E-05**	*****
Residuals	88	44	0,5			

Table S2. TukeyHSD post hoc pariwise analysis of interaction effects between biofilm and planktonic and (A) colistin treatment during evolution and (B) growth mode, colistin treatment and PAP concentrations.

Growth Mode	diff	p adj	significant
**PL-BF**	**-0,7**	**5,10E-07**	*****

Growth Mode: Treatment	evolution	diff	p adj	significant
PL-BF	0.5mg/L	-0,667	0,177	
PL-BF	0mg/L	1,78E-15	1	
PL-BF	1mg/L	-0,8	0,0509	
**PL-BF**	**8mg/L**	**-1,33**	**0,0000398**	*****

Growth Mode:PAP:Treatment	evolution	PAP	diff	p adj	significant
PL-BF	0mg/L	4 mg/L	-3,77E-15	1	
PL-BF	0mg/L	8 mg/L	3,55E-15	1	
PL-BF	0mg/L	16 mg/L	1,78E-14	1	
PL-BF	0mg/L	32 mg/L	3,55E-15	1	
PL-BF	0.5mg/L	4 mg/L	-8,88E-16	1	
**PL-BF**	**0.5mg/L**	**8 mg/L**	**-2,33**	**0,0336**	*****
PL-BF	0.5mg/L	16 mg/L	-1	0,997	
PL-BF	0.5mg/L	32 mg/L	-3,55E-15	1	
PL-BF	1mg/L	4 mg/L	-3,77E-15	1	
PL-BF	1mg/L	8 mg/L	-1	0,997	
**PL-BF**	**1mg/L**	**16 mg/L**	**-3**	**0,000545**	*****
PL-BF	1mg/L	32 mg/L	-3,55E-15	1	
PL-BF	8mg/L	4 mg/L	-4,44E-16	1	
PL-BF	8mg/L	8 mg/L	-0,333	1	
**PL-BF**	**8mg/L**	**16 mg/L**	**-4,67**	**1,89E-09**	*****
PL-BF	8mg/L	32 mg/L	-1,67	0,516	

Table S3. ANOVA of resistance frequency at day 10 for biofilm and planktonic (growth mode), colistin experimental treatments (0 mg/L – No Treatment, 0.5 mg/L- Static, 1 mg/L- Static, and 0.5 to 8 mg/L- Increasing), and PAP colistin concentrations (4 mg/L, 8 mg/L, 16 mg/L, and 32 mg/L).

	Df	Sum Sq	Mean Sq	F value	Pr(>F)	
Growth Mode	1	0,043	0,0428	0,606	0,4385	
**PAP**	3	**9,182**	**3,0608**	**43,357**	**<2E-16**	*
**Treatment**	3	**0,878**	**0,2926**	**4,144**	**0,0085**	*
Growth Mode:PAP	3	0,034	0,0113	0,16	0,9229	
Growth Mode:Treatment	3	0,043	0,0145	0,205	0,8926	
PAP:Treatment	9	0,437	0,0486	0,688	0,7176	
Growth Mode:PAP:Treatment	9	0,096	0,0107	0,151	0,9978	
Residuals	88	6,212	0,0706			

Table S4. Genomic location of mutations associated with colistin resistance in *P. aeruginosa* biofilm and planktonic populations. Columns represent the size, type and location, colistin treatments during evolution (No treatment, static and increasing), growth modes (biofilm – planktonic) and colistin concentration in PAP plates for harvest (8 and 32 mg/L)

Genomic location	Growth mode	0 mg/L - No Treatment	0.5 mg/L - Static	1 mg/L - Static	0.5 to 8 mg/L - Increasing
Nodulation protein NfeD	Biofilm	A162A (GCG→GCT)			
Nodulation protein NfeD	Planktonic	A162A (GCG→GCT)	A162A (GCG→GCT)	A162A (GCG→GCT)	A162A (GCG→GCT)
PhoP/Q and low Mg2inducible outer membrane protein H1	Biofilm			W63* (TGG→TGA) , coding (140/603 nt)	W63* (TGG→TGA) , Q69* (CAG→TAG)
PhoP/Q and low Mg2inducible outer membrane protein H1	Planktonic				Q70* (CAG→TAG)
Sensor protein QseC	Biofilm	coding (994/1434 nt)	R429H (CGC→CAC) , T132P (ACC→CCC) , coding (132-134/1434 nt), coding (507-509/1434 nt)	R429H (CGC→CAC)	T132P (ACC→CCC) , S166T (TCG→ACG) , P254S (CCG→TCG)
Sensor protein QseC	Planktonic	W134R (TGG→AGG)	I295L (ATC→CTC) , L90Q (CTG→CAG)	V285A (GTC→GCC) , V136L (GTG→TTG)	I295L (ATC→CTC) , L90Q (CTG→CAG) , R155H (CGC→CAC)
Virulence sensor histidine kinase PhoQ	Biofilm		V260G (GTC→GGC) , coding (779/1347 nt)	coding (1061/1347 nt), S224* (TCG→TAG)	coding (1061/1347 nt), V260G (GTC→GGC)
Virulence sensor histidine kinase PhoQ	Planktonic		V260G (GTC→GGC) , coding (134-144/1347 nt), V382G (GTC→GGC)	coding (134-144/1347 nt), V382G (GTC→GGC) , coding (627-631/1347 nt)	coding (134-144/1347 nt), V382G (GTC→GGC)

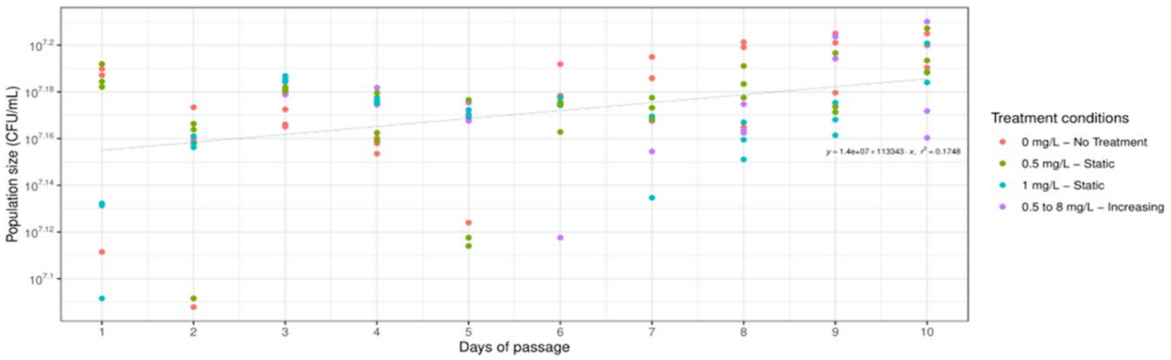

Figure S1. Overview of population size (CFU/mL; equivalent to CFU/bead) at each day of passage, for different colistin drug treatment conditions.

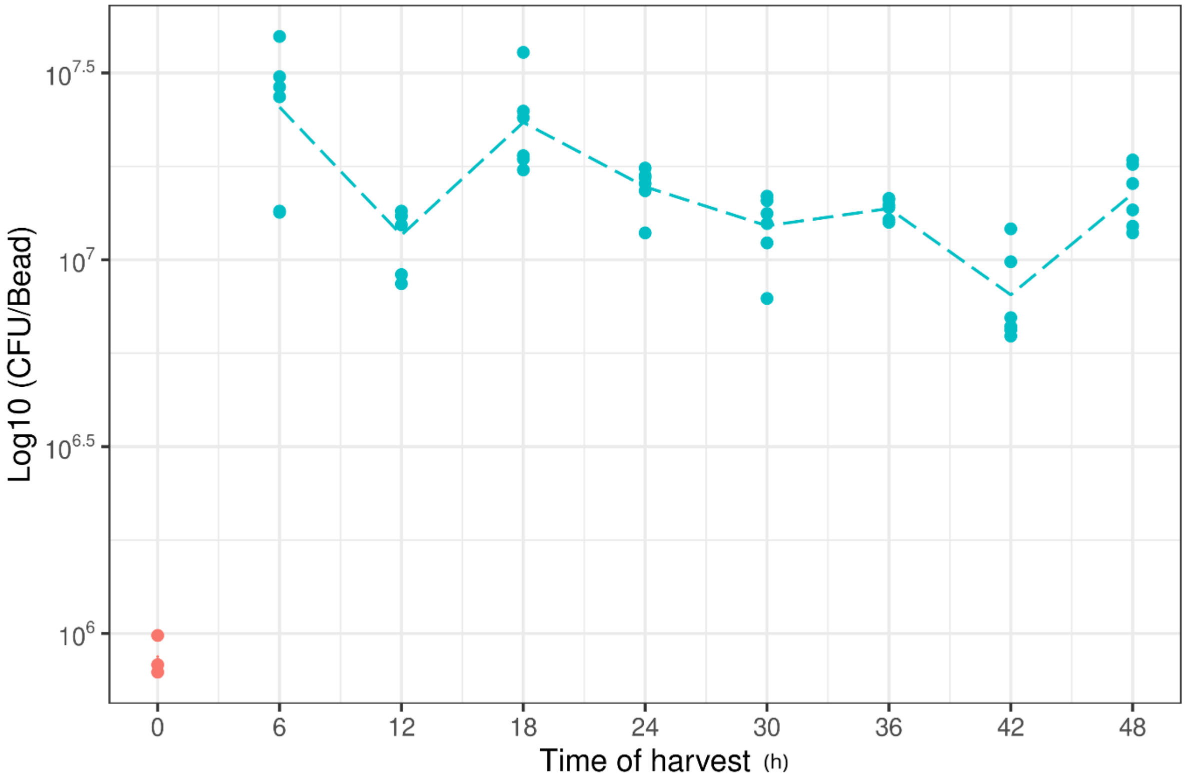

Figure S2. Overview of population size (CFU/bead) at different times of culturing.

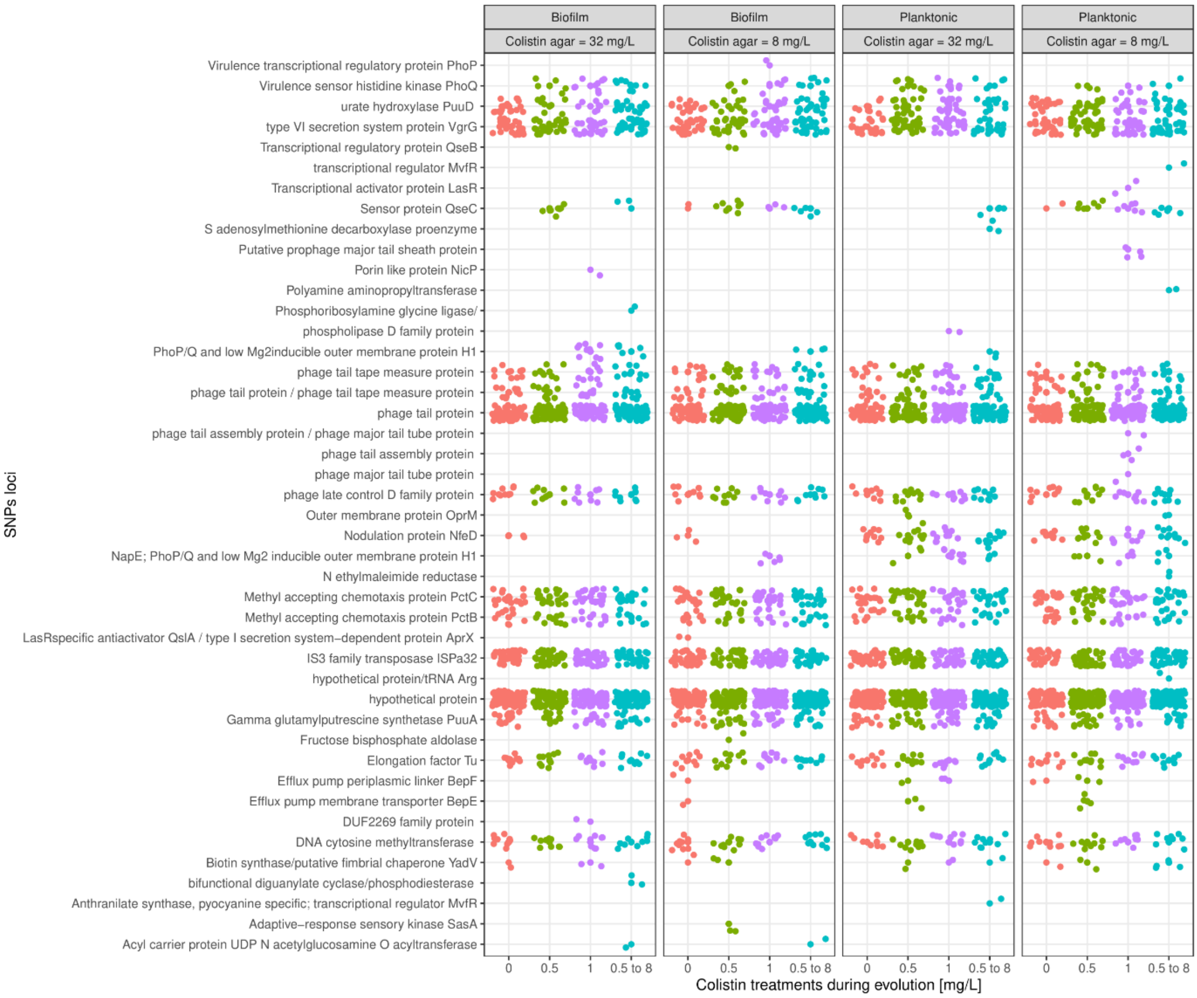

Figure S3. Overview of genes and hypothetical proteins for which SNPs were identified for isolates terminal samples from the evolution study, for different growth modes, colistin concentration conditions, and resistance levels (PAP, 8 and 32 mg/L).

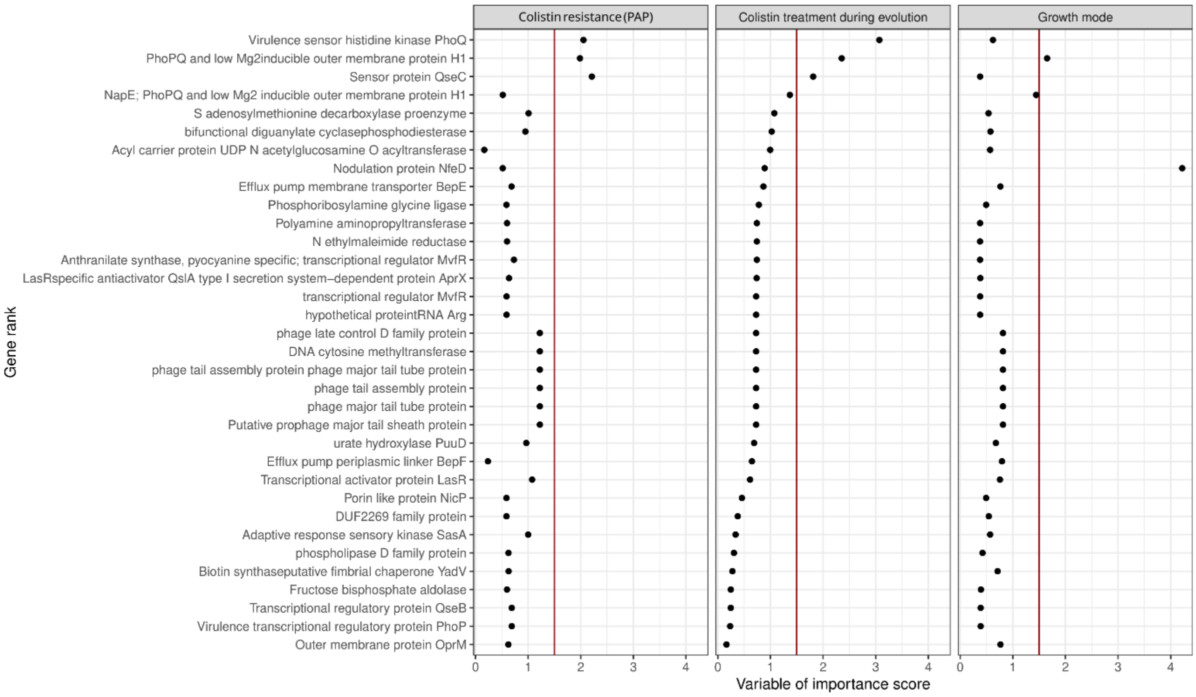

Figure S4. Variable importance scores derived from a PLS-DA analysis per gene for which SNPs were identified for terminal samples in the evolution experiment, with discriminatory variables: colistin resistance (PAP, 8 vs 32 mg/L), colistin treatment condition, and growth mode (planktonic or biofilm).

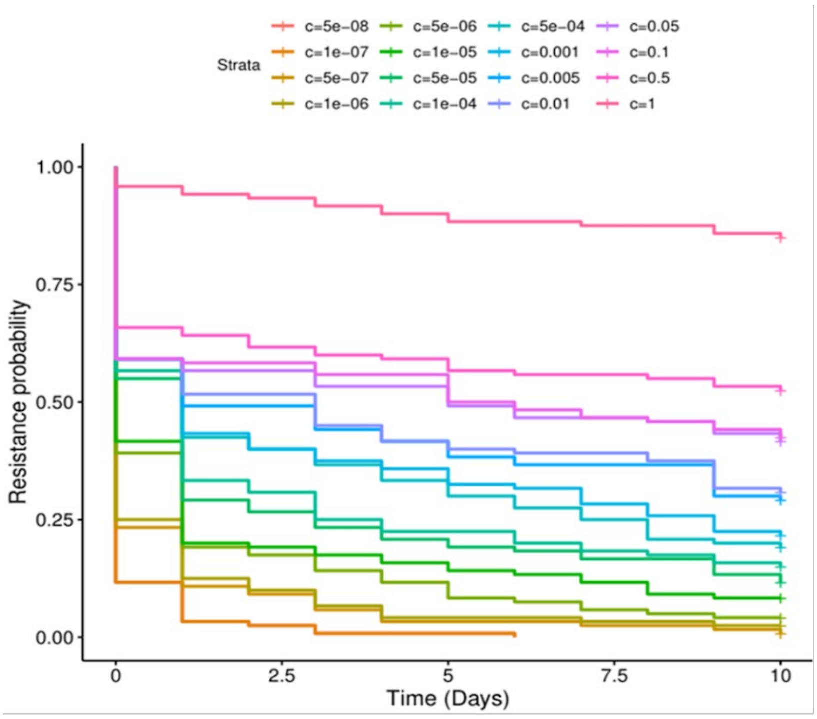

Figure S5. Kaplan–Meier analysis of resistance emergence across survival frequency thresholds. Each line represents the probability of detecting a resistant event (y-axis) over time (x-axis, in days), calculated from PAP data across all experimental conditions (biofilm and planktonic lifestyles combined). Colors correspond to different survival frequency thresholds, ranging from 5×10^{-8} to 1 (as indicated in the legend).

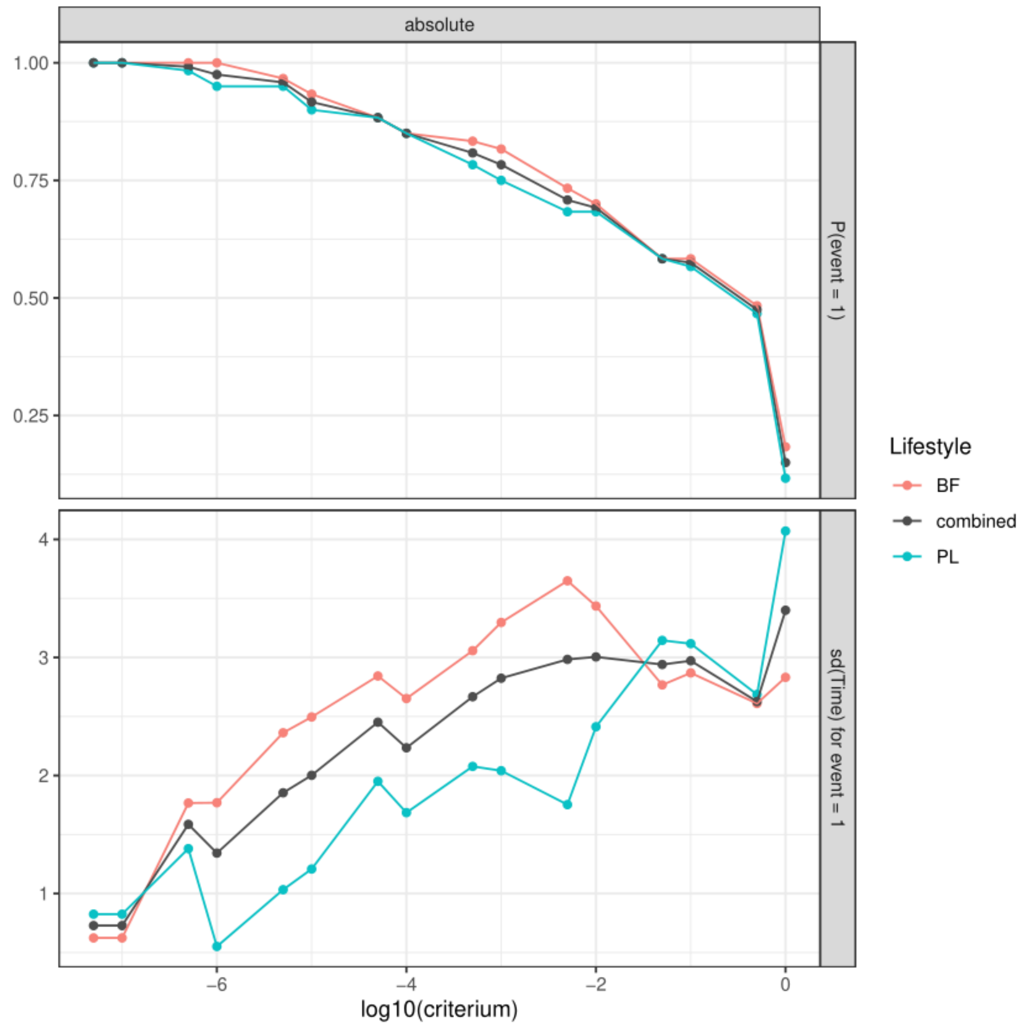

Figure S6. Probability and variability of resistance event detection across survival thresholds in PAP assays. Top panel: The y-axis shows the proportion of populations classified as based on varying survival frequency thresholds (x-axis, log_{10}-transformed). Bottom panel: The y-axis shows the standard deviation in the day of resistance event detection across replicates for each threshold. Thresholds range from 10^{-7} to 1 (x-axis, log_{10}), representing increasingly stringent cut offs of resistance.Lines represent biofilm (red), planktonic (blue), and both lifestyles combined (black).
